# Barriers and enablers to integrating physical activity in breast cancer care: A qualitative study using the TDF and COM-B model

**DOI:** 10.1007/s00520-026-10469-5

**Published:** 2026-03-02

**Authors:** Nathalie Piazzon, Florence Carrouel, Annick Gérard, Audrey Ringot, Marion Cortet, Elise Verot

**Affiliations:** 1 Health, Systemic, Process (P2S), UR4129, University Claude Bernard Lyon 1, Lyon, France; 2 Service de Gynécologie-Obstétrique, Hôpital de la Croix-Rousse, Hospices Civils de Lyon, Lyon, France; 3 Centre de Coordination en Cancérologie, Hôpital Lyon Sud, Hospices Civils de Lyon, Lyon, France; 4 LabTAU, INSERM U 1032, University Claude Bernard Lyon 1, Lyon, France; 5 CIC EC 1408 INSERM Saint-Etienne, Saint-Etienne, France; 6 PRESAGE Institut, University Jean Monnet, University of Lyon, Saint-Etienne, France

**Keywords:** Breast cancer, Physical activity, Qualitative study, Implementation, Nursing science, Theoretical Domains Framework

## Abstract

**Aims:**

This study aimed to identify the behavioral determinants influencing the integration of physical activity into the care pathway of postmenopausal women with hormone receptor–positive breast cancer. It specifically addressed the following research question: What are the main barriers and facilitators, from both patient and healthcare professional perspectives, that influence the integration of physical activity into routine care?

**Methods:**

This qualitative study was based on the Theoretical Domains Framework and the Capability, Opportunity, Motivation – Behavior (COM-B) model.

Semi-structured interviews were conducted and the analysis was guided by these frameworks, complemented by inductive thematic analysis, to capture nuanced insights in this clinical context.

**Results:**

Barriers for patients include misconceptions, fatigue and difficulties with autonomous PA practice, while healthcare professionals emphasize the need for practical training and clear protocols. Key enablers included early physical activity assessment, personalized and playful interventions, peer support, and the emerging role of advanced practice nurses in structuring care transitions. Digital tools show promise for sustainable engagement when co-designed with patients and integrated into therapeutic relationships.

**Conclusion:**

The study identifies concrete levers for integrating physical activity into oncology care, combining behavioral frameworks with nursing science to better understand clinical realities. These findings provide practical guidance for developing sustainable strategies and reinforce the need for person-centered, coordinated approaches to make physical activity a fully recognized component of breast cancer care.

**Supplementary Information:**

The online version contains supplementary material available at 10.1007/s00520-026-10469-5.

## Introduction

Breast cancer remains the most diagnosed cancer among women globally, representing one in eight cancer cases. Annually, 2.3 million new cases are reported across both sexes [1].

Advances in early detection and treatment have markedly improved outcomes, with five-year survival now reaching 90% in the United States [2]. Hormone receptor–positive breast cancers which constitute approximately 80% of cases, are typically diagnosed at a median age of 63 years [3].

In postmenopausal women with hormone receptor-positive breast cancer, aromatase inhibitors (AI) are the standard adjuvant hormonal therapy [4]. However, approximately 46% of patients experience, musculoskeletal symptoms, collectively termed Aromatase Inhibitor-Induced Musculoskeletal Syndrome (AIMSS). This condition adversely impacts quality of life and treatment adherence [5].

Managing AIMSS requires an integrated approach combining pharmacological and supportive care interventions. Among the supportive interventions, regular physical activity (PA) is associated with numerous benefits. PA is linked to improved overall and disease-specific survival and a reduced risk of recurrence [6, 7]. It also mitigates fatigue [8] and enhances quality of life [9]. While high-level evidence for PA in managing AIMSS remains limited, emerging studies suggest that adapted PA programs may relieve associated joint pain [10].

Despite the benefits of PA in oncology, its implementation remains limited. Among the women with breast cancer, low physical activity levels are linked to individual, social and organizational barriers [11]. To address these, Schmitz et al. [12] proposed a dual approach focused on both patient behavioral and healthcare professional practices. However, the available qualitative studies most often focus either on the experiences of patients [13, 14] or on those of healthcare professionals [11, 15], without providing an integrated analysis of both perspectives. This fragmentation limits the understanding of the interactional dynamics that shape the effective implementation of PA within care pathways. Additionally, while many studies have demonstrated the efficacy of PA interventions, their implementation in real-world oncology remains limited [16]. Fewer than half of evidence-based cancer care interventions are adopted in practice [17], highlighting the need for implementation science approaches [18]. Frameworks such as those proposed by Proctor et al. [19] emphasize adoption, sustainability, and effectiveness, with behavior change as a key mechanism to support long-term adherence.

Within this context, behavior change techniques (BCTs) provide a well-established approach for analyzing and designing targeted interventions [20].

To examine these behavioral dynamics, robust conceptual frameworks from the behavioral sciences are essential. Among these, the COM-B model (Capability, Opportunity, Motivation – Behavior) and the Theoretical Domains Framework (TDF) are particularly well-suited. The COM-B model identifies the conditions necessary for adopting of a specific behavior [21], while the TDF offers a comprehensive framework for exploring behavioral determinants, encompassing 14 domains derived from 33 behavior change theories [22]. Together, these frameworks provide a systemic perspective for identifying barriers and facilitators to integrating PA into clinical care [23].

Finally, no study to date has examined in depth the role of advanced practice nurses or drawn on nursing theories to inform the integration of physical activity into care pathways, even though these approaches could strengthen patient support, coordination, and continuity of care.

This study seeks to investigate the behavioral determinants shaping the integration of PA into the care pathway of postmenopausal patients treated for hormone receptor-positive breast cancer, drawing on the perspectives of both patients and healthcare professionals, using the TDF and the COM-B model. It aims to identify the barriers and facilitators influencing the adoption of PA, from the perspectives of both patients and healthcare professionals. These findings will inform the development of targeted interventions to promote the sustainable integration of PA into routine breast cancer care.

## Methods

This qualitative study followed the COREQ (Consolidated Criteria for Reporting Qualitative Research) guidelines, in accordance with the recommendations of the EQUATOR (Enhancing the Quality and Transparency of Health Research) [24].

Semi-structured interviews were conducted until data saturation was achieved. Data collection, carried out progressively and in parallel with the analysis, enabled determination of the final sample size [25].

### Population

The qualitative study was conducted in XXX at two sites: the gynecological surgery departments of Saint-Etienne University Hospital and Croix-Rousse University Hospital.

Investigating physicians used purposive sampling to identify eligible patients during routine consultations, selecting individuals who met predefined criteria while ensuring maximum variation in characteristics such as age, clinical status, and PA history [26]. Although clinicians were aware of patients’ backgrounds, they were instructed not to preferentially approach only highly active or motivated individuals, to avoid over-representing patients already engaged in PA. Healthcare professionals were also recruited using a purposive approach to ensure diversity in roles and levels of experience. All participants received standardized written and oral information and were included on a non-opposition basis. The potential for selection bias inherent to clinician-led recruitment was acknowledged when interpreting the results.

Eligible patients were (1) females aged between 35 and 75 years, (2) diagnosed with non-metastatic breast cancer, (3) positive for at least one hormone receptor, (4) undergoing treatment with an aromatase inhibitor and (5) affiliated with a social security system.

Eligible healthcare professionals were those involved in the routine care of breast cancer patients. They were recruited from various departments. The number and composition of professionals interviewed varied slightly across sites, depending on availability and the local organization of care teams.

### Theoretical framework

The TDF, initially structured around 12 domains, was revised to produce an enriched version comprising 14 domains encompassing 84 theoretical constructs derived from 33 behavioral theories [22, 23].

It offers a comprehensive and systematic view of the cognitive, affective, social and environmental determinants that influence individual behaviors and professional practices [27]. It helps structure the collection and analysis of qualitative data around clearly defined and validated factors.

By providing a clear structure, the TDF has established itself as a strategic tool for understanding the behavioral mechanisms involved in the adoption of or resistance to clinical recommendations [22], particularly in the field of physical activity [23].

The COM-B model was used as an analytical tool to facilitate the organization and interpretation of the results [21] (see Table 1). It is based on the principle that behavior results from the dynamic interaction between capability, opportunity and motivation. Combined with the TDF, the COM-B model enriches the analysis by grouping qualitative data around central behavioral mechanisms, while facilitating the structured identification of barriers and facilitators to the integration of PA into care [21–23].

**Table 1:**
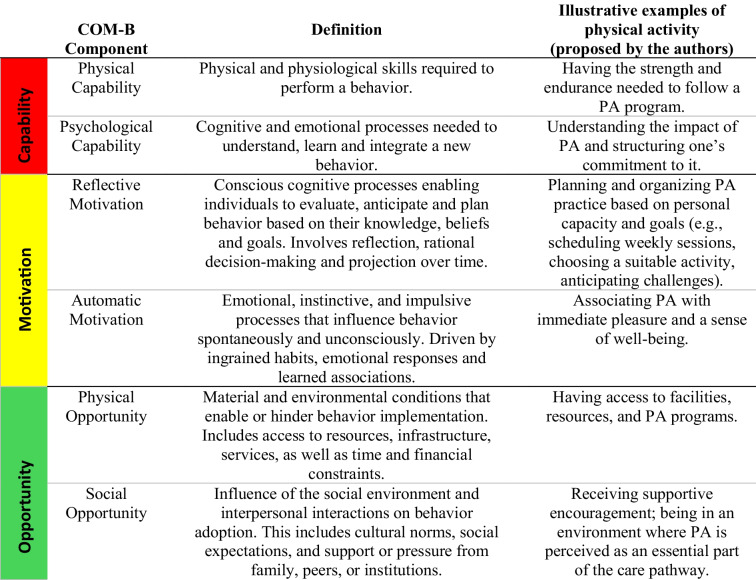
COM-B Model: Components and definitions, original and adapted table based on the work of Michie et al. [21], with illustrative examples of physical activity proposed by the authors

Abbreviations: *PA*, Physical Activity

**Fig. 1:**
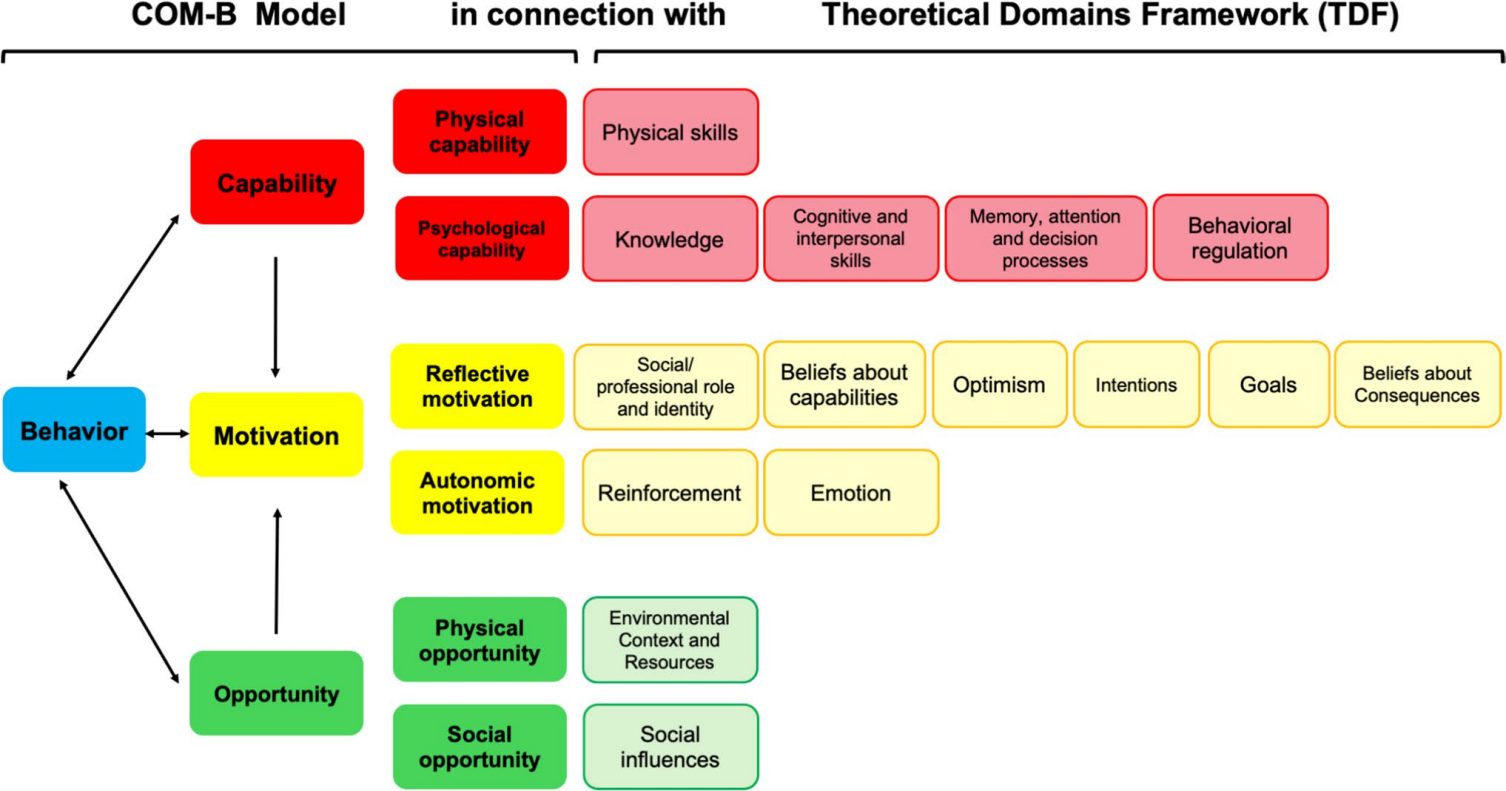
Links between the TDF and the COM-B model, based on Michie et al. [21]. Abbreviations: COM-B: Capability, Opportunity, Motivation-Behavior; TDF: Theoretical Domains Framework

Figure 1 illustrates their systematic links to the 14 domains of the TDF. This complementarity supports a structured transition from detailed analysis to an integrated understanding, helping to identify key barriers and levers for action based on individual profiles and contexts [21].

### Justification for the selection of TDF domains

This study adopts a comprehensive approach to exploring behavioral determinants related to PA integration. All 14 TDF domains were examined to ensure analytical rigor and comprehensiveness. A selection of the most relevant domains was then made, not based on their frequency of appearance, but on their interpretive value, following Braun and Clarke [28] principle of analytical relevance in thematic analysis.

Finally, from an intervention development perspective, as outlined in the Behavior Change Wheel model [26], the targeted selection of specific TDF domains can facilitate the practical translation of results. Thus, the findings from this study can serve as a valuable foundation for future work on the development of structured interventions.

### Data collection procedure

Two separate semi-structured interview guides, one for patients and the other for healthcare professionals, were developed based on the 14 domains of the TDF, following Cane et al. [22]. Each domain was addressed with an open-ended question, supported by follow-up prompts when needed [23].

The guides were reviewed and validated by two oncology healthcare professionals and a methodologist to ensure their relevance for the target audience. They were also pilot tested with a patient partner and a healthcare professional, leading to improvements for wording and clarity.

Interviews were conducted by a doctoral student who is also an advanced practice nurse (NP) and a patient researcher (AG), both trained in qualitative research. All participants received written and verbal information about the study, including its objectives, the interview process and data management.

Each interview lasted between 25 and 35 min (average: 31 min) and was conducted via a single videoconference session. With the participants’ consent, interviews were audio-recorded using a digital device, securely transferred to a computer and then deleted from the recorder. Transcripts were produced verbatim and pseudonymized. Data were organized and analyzed using NVivo® 15 software (QSR International).

### Ethics

The study protocol was approved by the local ethics committee of the Hospices Civils de Lyon (France) on 22 June 2023 (approval no. 2023–06–16). All participants received both written and oral information about the study. They gave their informed consent.

### Data analysis

Data were analyzed using a combined approach, involving both a deductive analysis based on the theoretical framework of the TDF and an inductive thematic analysis [23]. This process adhered to established methodological standards for qualitative research, ensuring rigor, transparency and reproducibility [29].

The first step of the analysis involved deductively coding the transcripts using the 14 TDF domains as coding nodes [22, 23]. This approach structured the initial analysis by assigning segments of discourse to one or more theoretical domains, thereby facilitating the identification of behavioral determinants.

In the second step, these determinants were grouped according to the COM-B model, providing an integrated overview of the capability, opportunity and motivation factors influencing behavior [21, 23].

In the third step, an inductive thematic analysis was conducted to identify themes not covered by the initial theoretical framework. This phase was carried out following the six-phase approach well described by Braun and Clarke [28]. Inductive codes were added to capture key elements from participants’ interviews that were not described by the TDF domains. These new codes were then combined with those from the previous phases to construct interpretative themes and sub-themes, labelled with clear and representative titles. Although the analysis drew on all 14 TDF domains, only five were retained in the results table due to their direct interpretive value for addressing the research question and informing practical implications. The assessment of interpretive value followed established guidance in qualitative and behavioral science, which recommends prioritizing domains that demonstrate salience, i.e., recurrence and prominence across participants [28], explanatory power for understanding behavioral mechanisms [23], and relevance for designing interventions within the Behavior Change Wheel framework [30].

Finally, a participant validation process was conducted using a summary feedback format. No disagreements were reported, and no changes were made to the findings.

## Results

### Participant characteristics

Table 2 describes the characteristics of participants. A total of 34 participants were included in the study: 16 patients and 18 healthcare professionals. Patients were aged between 48 and 72 years, with a mean age of 60. Their adjuvant treatment varied. One patient had only AI, seven had radiotherapy and AI, six had and AI and two had chemotherapy, targeted therapy, radiotherapy and AI. The healthcare professionals encompassed a broad spectrum of disciplines involved in breast cancer care, including sports medicine physicians (*n* = 2), medical oncologists (*n* = 2), surgeons (*n* = 4), head nurses (*n* = 1), advanced practice nurses (*n* = 3), physiotherapists (*n* = 2), an adapted physical activity instructor (*n* = 1), and care coordinator nurses (*n* = 3). Their clinical experience varied from 1 to 20 years, highlighting the diversity of professional backgrounds.

**Table 2:**
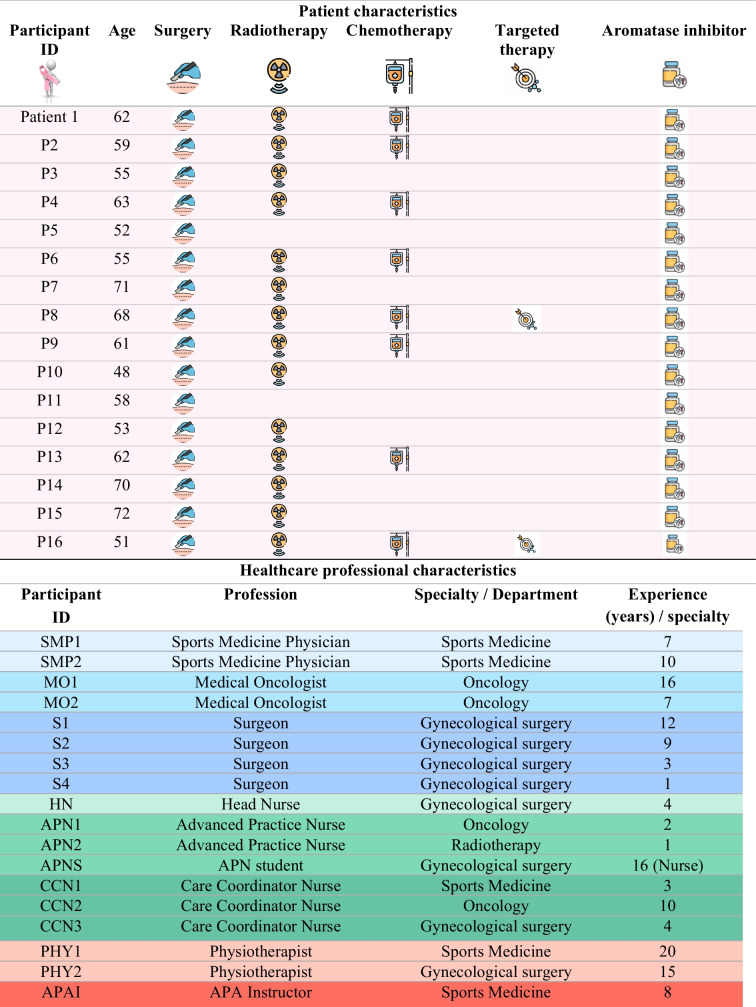
Characteristics of participants

Abbreviations: *APAI*, Adapted Physical Activity Instructor; *APN*, Advanced Practice Nurse; *APNS*, Advanced Practice Nurse Student; *CCN*, Care Coordinator Nurse; *HN*, Head Nurse; *ID*, identifier; *MO*, Medical Oncologist; *P*, patient; *PHY*, Physiotherapist; *S*, Surgeon; *SMP*, Sports Medicine Physician

Patients;  Physicians;  Nursing professionals;  Allied health professionals

### Identification of behavioral determinants: a thematic approach grounded in the COM-B and TDF models

The thematic analysis led to the identification of four main themes. Two related to individual factors linked to the patients (Determinants of resistance to PA, Determinants of patient engagement in PA. The other two themes (Restructuring of the care pathway and Underuse of digital tools) reflect organizational dynamics within the healthcare system. Each theme is broken down into several sub-themes, thirteen in total.

Table 3 presents an integrated synthesis of the major themes and sub-themes, their mapping to COM-B components and TDF domains and the associated codes.

**Table 3:**
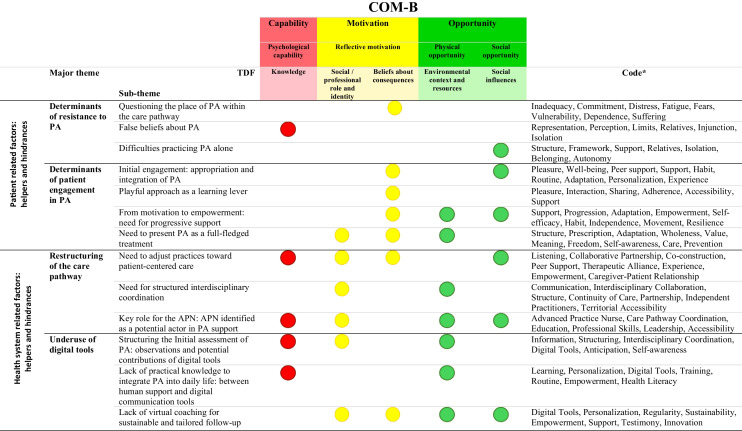
Overview and mapping of emergent themes, sub-themes and codes in relation to COM-B components and TDF domains

Abbreviations: *APN*, Advanced Practice Nurse; COM-B,  , Capability,  , Opportunity,  , Motivation Behavior; *PA*, Physical Activity; *TDF*, Theoretical Domains Framework

*Codes ranked in descending order of frequency and strength within the text

These themes were then mapped to the six components of the COM-B model (*physical capability*, *psychological capability*, *reflective motivation*, *automatic motivation*, *physical opportunity*, *social opportunity*) and the eleven domains of the TDF framework, to identify the behavioral determinants involved. Details of this mapping are available in Online Resource 1. Among the COM-B components, four emerged as particularly prominent in the analysis: psychological capability, reflective motivation, physical opportunity and social opportunity.

### Identification of barriers and facilitators based on behavioral determinants (COM-B/TDF)

The behavioral determinants identified through the COM-B and TDF offer a theoretical basis for understanding factors that support or hinder the adoption of PA. Detailed analysis of participant transcripts allowed for the translation of these determinants into concrete barriers and facilitators.

Table 4 outlines, for each sub-theme, the corresponding barriers and facilitators. These elements deepen the understanding of the factors influencing PA and complement the analytical mapping presented in Table 3.

This provides a meaningful foundation for identifying potential intervention functions.

### Patient-related barriers to PA

Several obstacles limit the integration of PA into care pathways. Many patients see it as an added burden in an already demanding physical and emotional context. Misconceptions, such as equating PA with intense sport, and limited awareness of its benefits reinforce this perception. Fatigue, fear of overexertion, and vulnerability further discourage engagement. The shift to autonomous practice often represents a critical phase. Following supervised sessions, patients frequently report a loss of structure, motivation or confidence. Feelings of isolation or discomfort in conventional fitness environments are also common.

### Patient-related facilitators and engagement strategies

Several facilitators support PA engagement, including pleasure, well-being, bodily reconnection and a sense of empowerment. Tailored accessible formats and personalized guidance foster ownership. Even light routines help sustain adherence. Positive experiences inspire patients to become peer supporters, motivated by a genuine desire to share their journey and help others. Playful, social formats, like group walks or games, promote engagement, especially among sedentary patients, by reducing effort-related barriers and enhancing motivation through peer interaction.

### Shared perspectives on integrating PA into care

Both patients and professionals’ express interest in implementing structured PA assessments early in the care pathway. From the perspective of healthcare professionals, early introduction of PA supports better treatment tolerance and more seamless integration into everyday life. However, diverging views emerge regarding the mode of integration: while some professionals voice concerns about potential drawbacks of systematic prescriptions, many patients advocate for PA to be treated as an essential component of care, comparable to pharmacological treatment.

### Organizational levers and structural challenges

Organizationally, early identification of needs and implementation of structured, stepwise support are key. The Advanced Practice Nurse (APN) role is a strategic lever for coordination, patient education and referral to adapted programs. This emerging role is also seen as a resource for training healthcare professionals, co-constructing objectives and ensuring continuity of support. A strong need for accessible information, training and structured guidance is reported by both groups. Healthcare professionals highlight the lack of practical tools for prescribing and supporting PA, while patients express a desire for clear, comprehensible information delivered throughout care.

### Digital tools: promises and limitations

The long-term integration of PA is hindered by a lack of coordination between hospital and community settings, limited visibility of existing resources and underuse of digital tools. Both patients and healthcare professionals suggest developing hybrid approaches combining human support and digital tools, to enable personalized, non-judgmental follow-up and facilitate sustainable integration of PA into daily life.

A comprehensive overview of the behavioral determinants, including barriers and facilitators, along with illustrative quotes from participants, is provided in Online Resource 2.

**Table 4:**
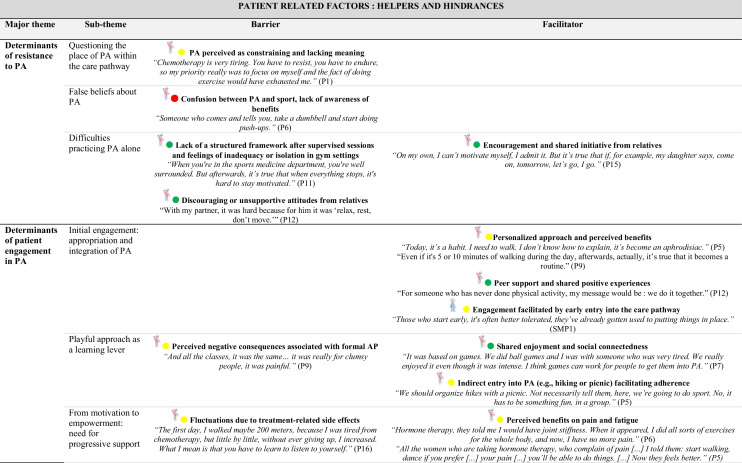

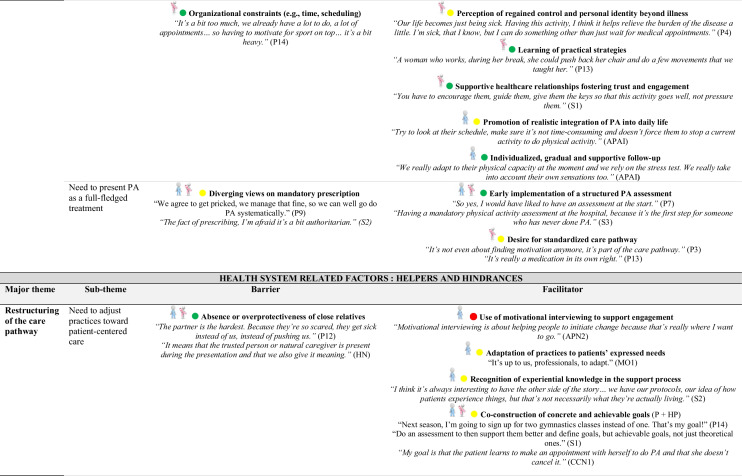

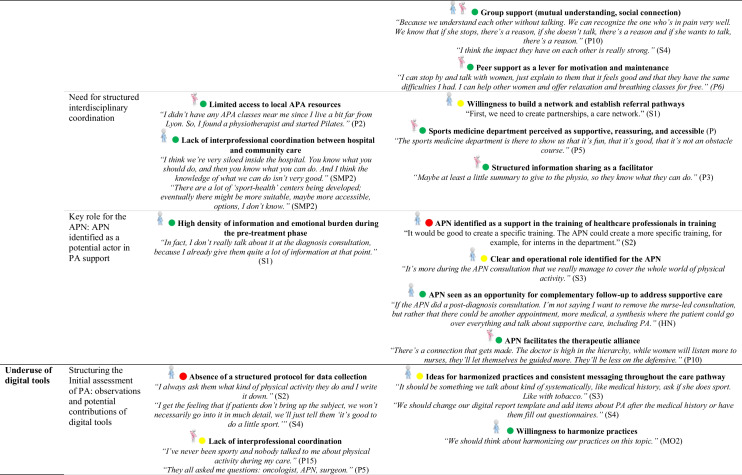

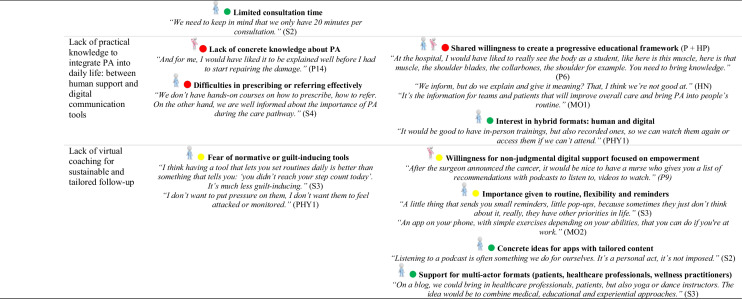
Identification of barriers and facilitators by sub-theme

Abbreviations: *APA*, Adapted Physical Activity; *APAI*, Adapted Physical Activity Instructor;

*APN*, Advanced Practice Nurse; *APNS*, Advanced Practice Nurse Student; *CCN*, Care Coordinator Nurse; *HN*, Head Nurse; *HP*, Healthcare Professional; *MO*, Medical Oncologist; *P*, Patient; *PA*, Physical Activity; *PHY*, Physiotherapist; *SPM*, Sports Medicine Physician; *S*, Surgeon

COM-B:  Capability,  Opportunity,  Motivation Behavior

Patient;  Healthcare Professional;  Patient and Healthcare Professional

**Fig. 2:**
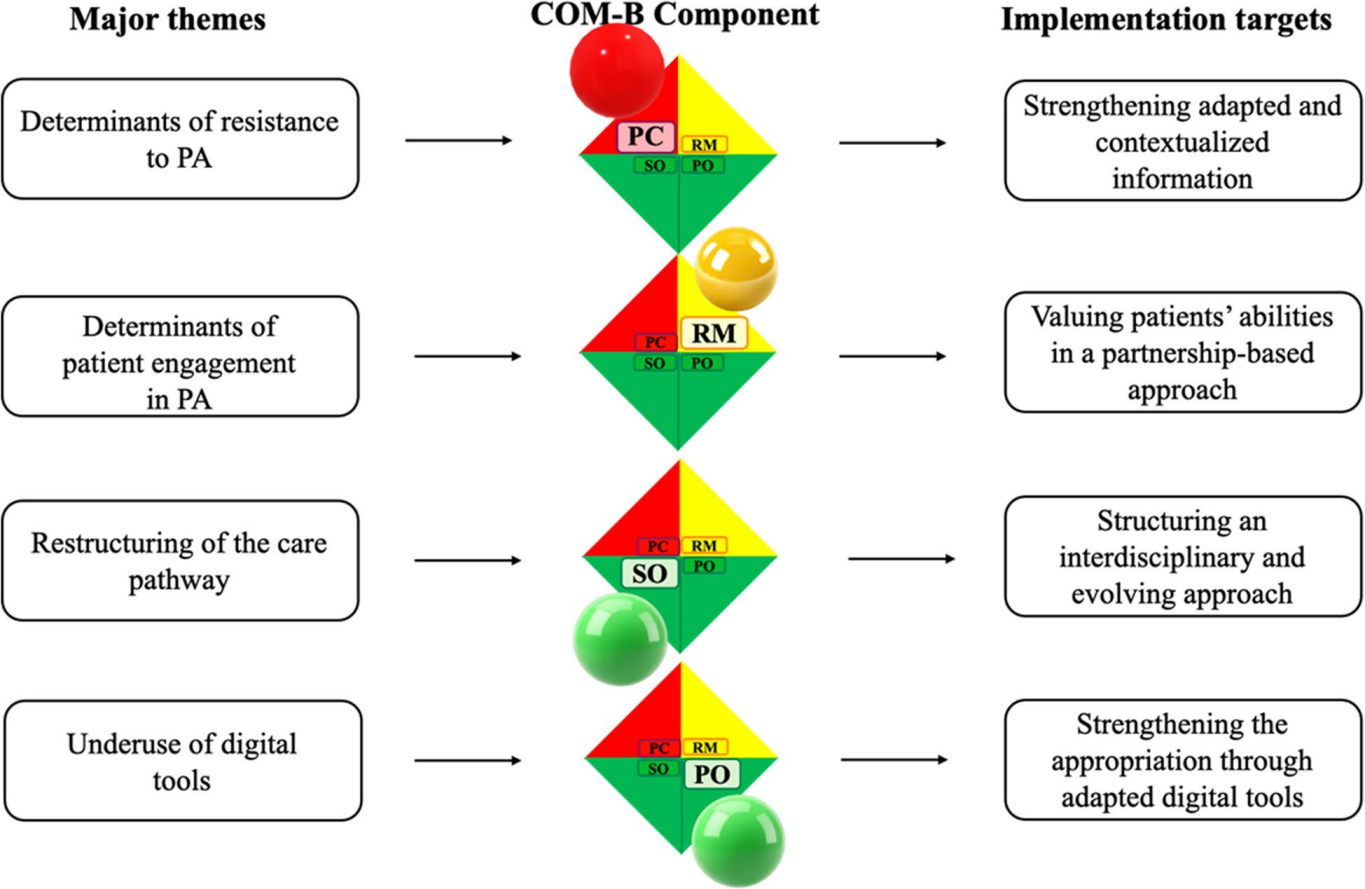
Mapping of the four major themes to COM-B determinants and corresponding implementation targets. Abbreviations: COM-B:  Capability,  Opportunity,  Motivation Behavior; PC: Psychological Capability; PO: Physical Opportunity; RM: Reflective Motivation; SO: Social Opportunity

To provide a concise synthesis of the behavioral mechanisms underlying these results, Fig. 2 presents an integrative diagram linking the four main themes to their corresponding COM-B determinants and related implementation objectives.

## Discussion

The findings provide an understanding of the obstacles and facilitators to PA engagement, from both patient and healthcare professional perspectives, and offer valuable insights into the mechanisms that could support sustainable implementation within a complex care setting.

### Strengthening adapted and contextualized information

The results indicate that information on PA is often perceived by patients as incomplete, poorly tailored, or disconnected from their lived experience. A frequent confusion between PA and athletic performance reinforces this gap, creating negative or unrealistic perceptions that discourage engagement. Lavallée et al. [13] emphasized the need for personalized information to support PA during breast cancer treatment. Such information should be embedded within an educational strategy that highlights perceived benefits, addresses misconceptions, and accounts for health literacy, which has been identified as a predictor of PA after treatment [14]. Moreover, many patients express a wish to receive information at the very beginning of their care pathway. Patient therapeutic education, which is usually introduced at the start of hormone therapy [31], could therefore be reconsidered to integrate behavioral components related to PA earlier in the trajectory.

Beyond informational gaps, the results highlight disease-specific fears that strongly influence patients’ engagement in PA. Several women expressed concerns about the risk of pain, increased fatigue, or overexertion, revealing a perception of bodily vulnerability that cannot be alleviated by information alone. These fears echo recent work showing that kinesiophobia, the fear that movement will aggravate symptoms, can significantly hinder physical activity among breast cancer survivors [32]. These observations underscore the need for educational strategies that, in addition to providing accurate information, help patients adjust their efforts, interpret bodily signals and regain confidence in their physical abilities.

From the healthcare professionals’ perspective, the main barriers reflect a lack of practical training, including how to prescribe, to whom to refer, and which tools to use. An ASCO workforce survey reported that 90% of oncologists felt insufficiently prepared to appropriately refer their patients to PA programs [33]. This knowledge gap, also described as an organizational barrier, highlights the need for simple referral protocols, targeted training, and the systematic integration of PA assessment into the medical record from the start of care to strengthen coordination and follow-up [34].

### Valuing patients’ abilities in a partnership-based approach

Engagement in PA largely depends on how patients perceive their ability to act and progress, despite treatment side effects or physical limitations. This study highlights several factors that support this process: enjoyment of movement, recognition of progress, individualized goal setting and social support, particularly among peers. In this context, physical activity was more readily adopted when framed as a playful and social experience rather than as a formal or performance-oriented exercise. Patients described these peer groups as places of solidarity, mutual recognition and normalization, where the shared experience of cancer created a sense of belonging. These environments helped women feel understood without having to justify themselves and played a key role in promoting self-confidence [35]. Other studies confirm the positive impact of feedback and peer observation on self-efficacy, a central lever for behavior change [13].

In addition to these motivational and relational mechanisms, our results also underscore an identity-related dimension of engagement in physical activity. Several women described physical activity to reconnect with a meaningful sense of self and regain continuity in their lives after the ordeal of cancer, echoing previous work highlighting identity reconstruction as a central component of adapting to illness [36]. This process, rarely discussed in exercise oncology, reinforces the importance of care approaches that acknowledge patients’ lived experience and support their efforts to restore a coherent sense of identity.

This perspective aligns with broader care models that engage all professionals along the care pathway. It reflects a clinical posture centered on the person, their lived experience and their strengths.

This approach is consistent with the theoretical foundations of Strengths-Based Nursing (SBN), developed by Laurie Gottlieb, which emphasizes resource mobilization and relational engagement in care [37].

SBN provides a structured framework for nurses to support patient empowerment, foster their involvement in care process and support long-term behavior change [38].

Although SBN was developed in nursing care, it can guide clinicians, educators, managers, and researchers, in creating work environments that promote interprofessional collaboration, patient empowerment, and sustainable interventions [39].

### Structuring an interdisciplinary and evolving approach

The results reveal organizational barriers such as fragmented coordination, absent systematic PA assessment and limited resource visibility. This is consistent with previous studies highlighting how professional silos hinder PA implementation [15].

Beyond these structural obstacles, the results reveal that clinicians are often hesitant to formalize their PA recommendations, not out of doubt about their relevance, but out of fear that structured prescriptions will be perceived as authoritarian or moralizing, an ethical concern rarely addressed in the exercise-oncology literature. This relational caution contrasts with the perspective of many patients, who consider PA an essential component of medical care, on par with pharmacological treatments.

Conversely, several facilitators appear repeatedly: the presence of identified reference persons (e.g., APN), access to structuring resources (e.g., sports medicine services) and the creation of shared tools. These facilitators not only improve patient orientation but also strengthen healthcare professionals’ sense of legitimacy in addressing PA, an element identified in the TDF as key to action initiation.

For patients, the results highlight several particularly challenging moments throughout their PA trajectory. These difficulties include starting the PA program, managing side effects such as fatigue or pain, and, above all, transitioning from supervised to independent practice. Several women described a significant decrease in their motivation, self-confidence and sense of direction after the end of structured support, which could be considered a behavioral change. Identifying these moments as critical transitions underscores the need to improve continuity of care pathways.

These intertwined individual and organizational transitions require a coordinating role capable of ensuring continuity of care, strengthening interdisciplinary collaboration and supporting patients during critical phases of their care pathway.

Considering all these elements, Meleis’s theory of transitions provides concrete guidelines for understanding and supporting these processes [40].

Within this framework, the APN emerges as a strategic actor in care transitions by adapting interventions to the evolving needs of patients and strengthening interprofessional collaboration [41]. This role, identified in this study as a potential lever, aligns with broader calls for stronger interprofessional collaboration and enhanced support during key phases of the patient journey [12, 40].

The application of this theory also provides insights into the mechanisms that act as levers for change: beyond task coordination, it is about creating a supportive care environment in which each actor, healthcare professional or patient, can adapt, learn and evolve in a spirit of partnership.

### Strengthening the appropriation through adapted digital tools

Patients express a need for simple, visual, and accessible tools to support understanding and engagement in PA. The aim is not complex e-health platforms, but useful aids (videos, interactive modules, apps, or podcasts).

Their benefits on health behaviors during cancer care are well documented [42].

Despite their potential, digital tools for PA remain underused in clinical practice, due to limited institutional integration and poor visibility of available resources [43]. Some professionals also express concern that prolonged use may induce stress or guilt, reflecting the ambivalent nature of self-tracking devices [44]. In addition, digital literacy appears to be a potential barrier, particularly for older adults who may feel anxious or lack confidence when using digital technologies [45]. However, recent data suggest that age alone is not a limiting factor: in a large randomized controlled trial, patients over the age of 65 demonstrated rates of adherence to digital symptom monitoring that were equal to or higher than those of younger participants, particularly when the tool was easy to use and accompanied by appropriate support [46]. These findings highlight that challenges related to digital literacy are modifiable and that personalized support can promote engagement, even among people with no prior digital experience.

These perceived limitations do not call into question the interest of digital tools but underline the importance of reflecting on their conditions of use.

Participants in this study emphasized that effectiveness depends on several conditions: co-designed content with patients, flexible use, and integration into a caring therapeutic relationship. Therefore, digital tools should not be considered as standalone devices, but as complementary supports, both to strengthen patient engagement and to support clinical practice.

Their progressive integration, at key moments in the care pathway, could help structure a shared culture around PA, in line with current recommendations on digital health literacy [47].

### Practical implications for the implementation of PA: towards targeted strategies

This study addresses key gaps identified in the literature by bringing together the perspectives of both patients and healthcare professionals within a unified behavioral framework (TDF—COM-B). This integrated approach makes it possible to move beyond general descriptions of barriers and facilitators and to identify operational levers directly relevant to clinical practice.

The contributions of Meleis’s and Gottlieb’s nursing theories enrich this perspective by introducing a dynamic dimension of care. These approaches also guide healthcare professionals by promoting a supportive, co-constructive, and coordinated posture, particularly suited to oncology pathways.

Together, these elements form a solid foundation for designing targeted, relevant and transferable interventions, guided by the Behavior Change Wheel and the APEASE criteria (Acceptability, Practicability, Effectiveness/cost-effectiveness, Affordability, Safety and Equity) [30].

### Limitations

This study has several limitations.

First, it was conducted at two hospital sites with mainly hospital-based participants. Further research is needed to assess the transferability of findings to other settings and populations.

Including community-based professionals, such as general practitioners or Adapted Physical Activity (APA) associations, could have added insight into coordination issues, resource access, and outpatient implementation barriers. A broader recruitment base may also reveal context-specific facilitators and obstacles not captured here.

Secondly, the diversity in healthcare professionals’ specialties and years of experience is likely to have shaped the nuances of their accounts, as professional roles, exposure to supportive care and institutional resources may influence how PA promotion is perceived and implemented. This variability is inherent to maximum variation sampling and contributed to capturing a wide range of organizational realities. At the same time, data saturation was reached, with convergent perspectives emerging across interviews, suggesting that the main themes reflected shared challenges and levers rather than institution-specific views. This potential influence of professional background should nevertheless be considered when assessing the transferability of our findings.

Thirdly, the findings are based on self-reported experiences. As in most qualitative designs, responses may be influenced by social desirability or recall bias, particularly among healthcare professionals.

Finally, despite collaborative coding and thematic triangulation, a degree of interpretive bias cannot be fully excluded.

Despite these limitations, the diversity of participant profiles and the richness of the data allowed for the identification of concrete, multidimensional levers to support the integration of adapted physical activity into the care pathway.

## Conclusion

This qualitative study contributes to laying the groundwork for a structured reflection aimed at developing sustainable implementation strategies that are both theoretically grounded and adapted to clinical practice realities. The findings also underscore the strategic role of APNs in supporting coordination and patient-centered care.

Rethinking PA through a strengths-based and partnership-oriented approach thus opens concrete avenues to support engagement, strengthen decision-making autonomy and sustainably establish PA as a fully recognized component of care.

## Supplementary Information

Below is the link to the electronic supplementary material.

Supplementary file1 (DOCX 245 KB)

Supplementary file2 (DOCX 50.5 KB)

## Data Availability

The interview transcripts generated and analyzed during the current study were pseudonymized and stored securely. In accordance with the information provided to participants regarding confidentiality, the datasets are not publicly available but may be obtained from the corresponding author on reasonable request.
