# Supplementary material for: Barriers and enablers to integrating physical activity in breast cancer care: A qualitative study using the TDF and COM-B model
Source: Support Care Cancer. 2026 Mar 2;34(3):261. doi: 10.1007/s00520-026-10469-5 (PMC12953373; doi:10.1007/s00520-026-10469-5)
Supplement: Supplementary file 1 — Supplementary file1 (DOCX 245 KB) [file 520_2026_10469_MOESM1_ESM.docx]

**Supplementary File 1** Mapping behavioral determinants of physical activity using the COM-B model and TDF

This supplementary file includes three complementary matrices. Each presents the alignment between inductively identified sub-themes and the theoretical components of the COM-B model and Theoretical Domains Framework (TDF). The first matrix (Part A) integrates perspectives from both patients and healthcare professionals. The second (Part B) focuses exclusively on patients and the third (Part C) on professionals. Together, they provide a structured and theory-informed interpretation of individual and organisational factors influencing physical activity (PA), supporting a comprehensive understanding of the behavioural mechanisms at play.


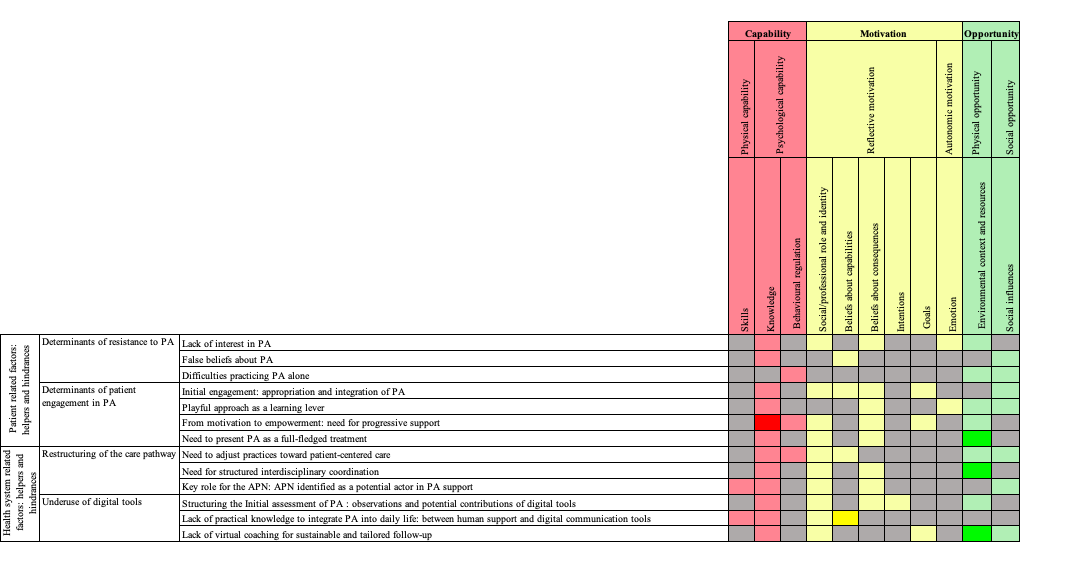


TDF Domains

Part A - Mapping behavioral determinants of PA in patients and healthcare professionals using COM-B and TDF

Capability component (COM-B) identified by both patients and healthcare professionals


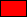


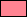
 Capability component (COM-B) by either patients or healthcare professionals


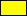
 Motivation component (COM-B) identified by both patients and healthcare professionals.


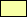
 Motivation component (COM-B) by either patients or healthcare professionals


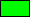
 Opportunity component (COM-B) identified by both patients and healthcare professionals


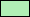
 Opportunity component (COM-B) by either patients or healthcare professionals

Among the COM-B components, four emerged as particularly prominent in the analysis ( ):

- Psychological capability, particularly in connection with the domain of *knowledge*
- Reflective motivation, associated with *beliefs about consequences* and *social/professional role and identity*
- Physical opportunity, related to *resources* and the *environmental context*
- Social opportunity, through support or barriers linked to the *social influences*


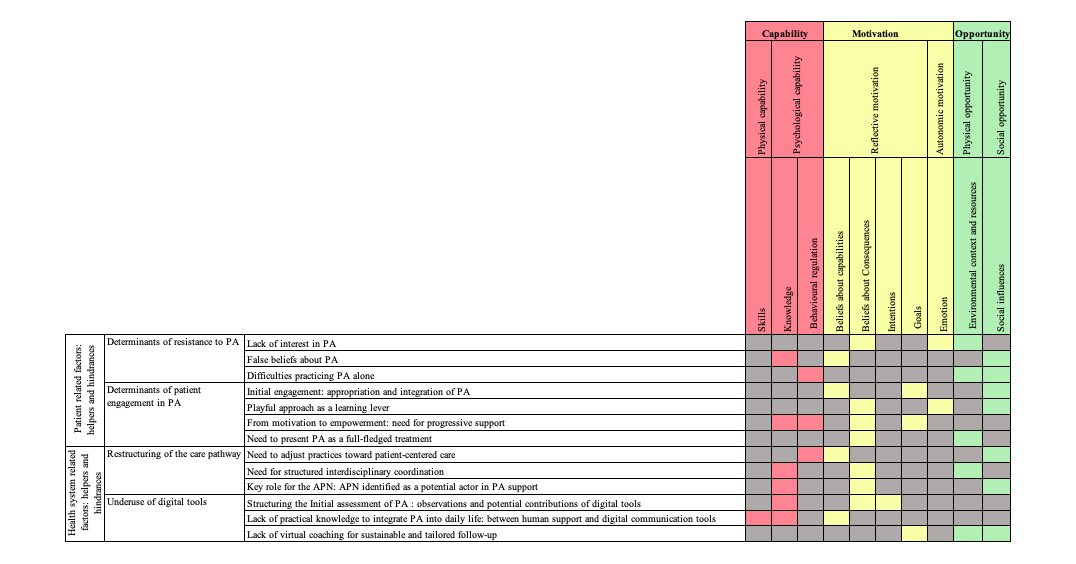


TDF Domains

Part B - Mapping behavioral determinants of PA in patients using COM-B and TDF


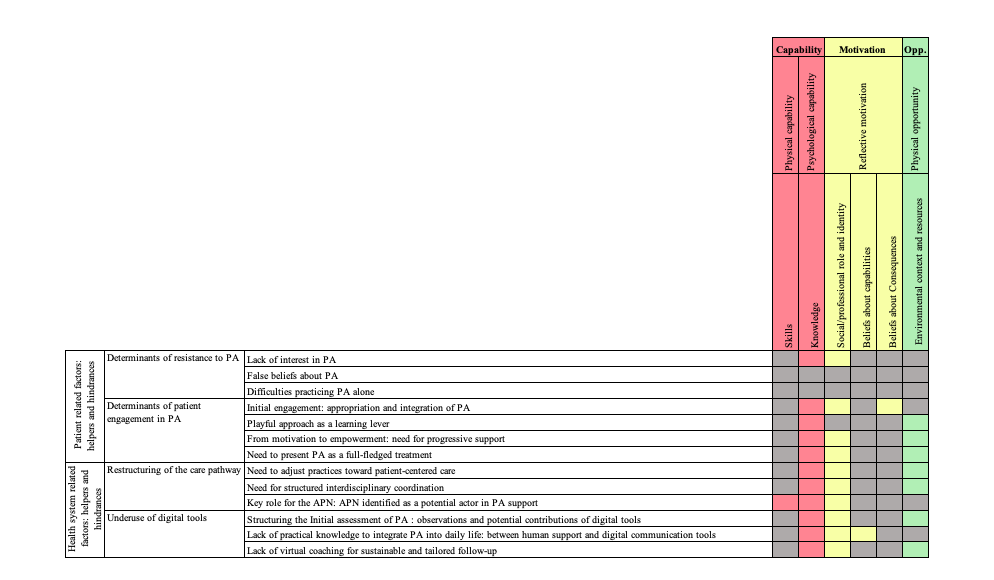


TDF Domains

Part C - Mapping behavioral determinants of PA in healthcare professionals using COM-B and TDF


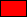
 Capability component (COM-B) identified by both patients and healthcare professionals


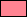
 Capability component (COM-B) by either patients or healthcare professionals


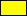
 Motivation component (COM-B) identified by both patients and healthcare professionals.


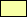
 Motivation component (COM-B) by either patients or healthcare professionals


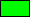
 Opportunity component (COM-B) identified by both patients and healthcare professionals


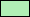
 Opportunity component (COM-B) by either patients or healthcare professionals
