# Supplementary material for: Barriers and enablers to integrating physical activity in breast cancer care: A qualitative study using the TDF and COM-B model
Source: Support Care Cancer. 2026 Mar 2;34(3):261. doi: 10.1007/s00520-026-10469-5 (PMC12953373; doi:10.1007/s00520-026-10469-5)
Supplement: Supplementary file 2 — Supplementary file2 (DOCX 50.5 KB) [file 520_2026_10469_MOESM2_ESM.docx]

**Supplementary File 2** Behavioural determinants of physical activity Integration: barriers and enablers perceived by patients and healthcare professionals

|  | **Major theme** | **Sub-theme** |
| --- | --- | --- |
| **Patient related factors: helpers and hindrances** | **Determinants of resistance to PA** | **Questioning the place of PA within the care pathway**  PA can be perceived as an additional constraint in a journey that is already heavy, both physically and emotionally.  *“It’s a bit too much, we already have a lot to do, a lot of appointments… so having to motivate for sport on top… it’s a bit heavy.”* (P14)  PA is sometimes associated with a repetitive effort, lacking meaning or pleasure.  *“When I say I’m fed up, really, I’m fed up, it’s too constraining.”* (P1)  Some patients describe a mismatch between their expectations and the proposed formats, such as sessions that are too standardised or not stimulating.  *“And all the classes, it was the same… it was really for clumsy people, it was painful.”* (P9) |
|  |  | **False beliefs about PA**  Patients hold inaccurate beliefs about PA. It can be perceived as an effort incompatible with their health condition, linked to a fear of not being physically capable.  *“I remember I was scared. I was scared there would be lots of movements I couldn’t do.”* (P11)  The fear of worsening treatment-related fatigue constitutes another major psychological barrier.  *“Chemotherapy is very tiring. You have to resist, you have to endure, so my priority really was to focus on myself and the fact of doing exercise would have exhausted me.”* (P1)  These fears are reinforced by a confusion between PA and technical or competitive sport.  *“Someone who comes and tells you, take a dumbbell and start doing push-ups.”* (P6)  PA is sometimes associated with an urban or institutional environment (gyms, specialized centers), perceived as inaccessible, particularly in rural areas.  *“Someone who’s never done sport, I think that around here, in the countryside, they’re screwed.”* (P3)  Loved ones may also convey discouraging representations out of protection.  *“With my partner, it was hard because for him it was ‘relax, rest, don’t move.’”* (P12) |
|  |  | **Difficulties practicing PA alone**  Engaging in PA independently appears to be a challenge for many patients. Two main factors related to feelings of isolation emerge from the verbatims: the absence of a structured framework after supervised sessions and a sense of inadequacy or isolation felt in gyms.  *“When you're in the sports medicine department, you're well surrounded. But afterwards, it’s true that when everything stops, it's hard to stay motivated.”* (P11)  *“Going to a gym, there's always this feeling of discomfort, of isolation. It’s the looks, the judgement, it's definitely not very pleasant.”* (P15)  Relatives are identified as a potential source of support, but also as a factor of inertia.  *“On my own, I can’t motivate myself, I admit it. But it’s true that if, for example, my daughter says, come on, tomorrow, let’s go, I go.”* (P15)  *“There are relatives who will encourage you, and others who will bring you down. ‘Oh! You’re doing sport now!’”* (P5) |
|  | **Determinants of patient engagement in PA** | **Initial engagement: appropriation and integration of PA**  For some patients, PA is a completely new experience.  *“There are ladies who discover sport with us, who buy their first pair of sneakers or leggings.”* (CCN1)  **Patient related factors: helpers and hindrances**  Entering PA relies on a personalized approach, respectful of everyone’s pace, and supported by simple, accessible formats.  *“Even if it's 5 or 10 minutes of walking during the day, afterwards, actually, it’s true that it becomes a routine.”* (P9)  *“To approach it, you need to be gentle and go case by case.”* (P3)  PA then becomes a way to reconnect with one’s body and a renewed sense of vitality.  *“A sense of well-being, we forget, we are in the present moment.”* (P2)  *“It also brings me inner peace and pleasure. That must be what PA is.”* (P10)  This experienced well-being motivates some patients to engage in peer support, sharing their positive experiences to encourage others who may hesitate.  *“For someone who has never done physical activity, my message would be: we do it together.”* (P12)  *“Of course, I won’t bring them knowledge, but I could share what it brought me.”* (P8)  *“All the women who are taking hormone therapy, who complain of pain, I met one and I told her: ‘go on, start walking, dance if you prefer. Take pleasure in doing it in the morning and you’ll see that your pain, little by little, it won’t go away completely, but you’ll be able to do things you wouldn’t even imagine’. She listened to my advice and now she feels better.”* (P5)  From the perspective of healthcare professionals, early initiation is seen as a key facilitator, helping to establish habits and improve treatment tolerance over time.  *“Those who start early, it's often better tolerated, they’ve already gotten used to putting things in place.”* (SMP1) |
|  |  | **Playful approach as a learning lever**  PA can be facilitated through a playful and friendly approach, particularly for sedentary patients.  *“It was based on games. We did ball games and I was with someone who was very tired. We really enjoyed it even though it was intense. I think games can work for people to get them into PA.”* (P7)  This playful dimension is often associated with the notion of shared pleasure, which helps to reduce the anxiety of “doing sport” in the traditional sense. By bypassing negative representations or effort-related barriers, these formats offer a first positive experience, conducive to adherence.  *“We should organize hikes with a picnic. Not necessarily tell them, here, we’re going to do sport. No, it has to be something fun, in a group.”* (P5) |
|  |  | **From motivation to empowerment: need for progressive support**  The transition toward autonomous PA practice is not linear. It fluctuates depending on treatment side effects or organizational constraints, but it becomes rooted when freely chosen.  *“The first day, I walked maybe 200 meters, because I was tired from chemotherapy, but little by little, without ever giving up, I increased. What I mean is that you have to learn to listen to yourself.”* (P16)  This process allows patients to progressively develop resilience, adaptability and self-regulation skills.  *“It took me a lot of time to find the right rhythm.”* (P3)  Progress depends on individualized adjustments and support from trained and attentive healthcare professionals.  *“We really adapt to their physical capacity at the moment and we rely on the stress test. We really take into account their own sensations too.”* (APAI)  *“You have to encourage them, guide them, give them the keys so that this activity goes well, not pressure them.”* (S1)  Over the course of their journey, patients gradually gain more freedom in their daily routines thanks to professional guidance.  *“A woman who works, during her break, she could push back her chair and do a few movements that we taught her.”* (P13)  *“Try to look at their schedule, make sure it’s not time-consuming and doesn’t force them to stop a current activity to do physical activity.”* (APAI)  This practice is supported by tangible benefits, such as better treatment tolerance.  *“Hormone therapy, they told me I would have joint stiffness. When it appeared, I did all sorts of exercises for the whole body, and now, I have no more pain.”* (P6)  Beyond physical effects, PA can become a space for identity reconstruction and empowerment.  *“Our life becomes just being sick. Having this activity, I think it helps relieve the burden of the disease a little. I’m sick, that I know, but I can do something other than just wait for medical appointments.”* (P4)  The initial intention of “doing something for oneself” progressively evolves into an integrated routine.  *“Today, it’s a habit. I need to walk. I don’t know how to explain, it’s become an aphrodisiac.”* (P5) |
|  |  | **Need to present PA as a full-fledged treatment**  Patients and healthcare professionals jointly express the wish to implement a structured PA assessment from the beginning of care.  *“So yes, I would have liked to have an assessment at the start.”* (P7)  *“Having a mandatory physical activity assessment at the hospital, because it’s the first step for someone who has never done PA.”* (S3)  While the value of early assessment is widely acknowledged, opinions diverge between patients and healthcare professionals regarding whether PA should be prescribed systematically.  Some patients believe that PA should be integrated into the care pathway like any other treatment, regardless of individual motivation.  *“We agree to get pricked, we manage that fine, so we can well go do PA systematically.”* (P9)  *“It’s not even about finding motivation anymore, it’s part of the care pathway.”* (P3)  *“It’s really a medication in its own right.”* (P13)  Conversely, healthcare professionals are more cautious about mandatory prescription, which they perceive as potentially counterproductive.  *“The fact of prescribing, I’m afraid it’s a bit authoritarian.”* (S2)  In short, integrating PA into the care pathway requires a structured organization based on personalized support, early screening coordinated by healthcare professionals and practice adaptation to reconcile patients’ expectations with healthcare providers’ perceptions. |
| **Health system related factors: helpers and hindrances** | **Restructuring of the care pathway** | **Need to adjust practices toward patient-centered care**  Adjusting practices is based on the professionals' adaptation to the needs expressed by patients.  *“It’s up to us, professionals, to adapt.”* (MO1)  *“I’m okay with being sick, but we’re not helpless, we’re not disabled. You still have to adapt the classes to the students.”* (P9)  The definition of personalised objectives, co-constructed between caregiver and patient, is presented as a lever to encourage engagement.  *“Next season, I’m going to sign up for two gymnastics classes instead of one. That’s my goal!”* (P14)  *“Do an assessment to then support them better and define goals, but achievable goals, not just theoretical ones.”* (S1)  *“My goal is that the patient learns to make an appointment with herself to do PA and that she doesn’t cancel it.”* (CCN1)  Motivational interviewing is also mentioned as a respectful support tool, allowing the patient to engage in a process of change that makes sense to her.  *“Motivational interviewing is about helping people to initiate change because that’s really where I want to go.”* (APN2)  Patients and healthcare professionals agree on the importance of the relational environment in the PA pathway. Three forms of support emerge: involvement of relatives, collective dynamics between patients and peer support.  The involvement of relatives, especially during information sessions, is seen as a potential lever to enhance their understanding of the issues related to PA and their ability to provide support.  *“The partner is the hardest. Because they’re so scared, they get sick instead of us, instead of pushing us.”* (P12)  *“It means that the trusted person or natural caregiver is present during the presentation and that we also give it meaning.”* (HN)  The collective dynamic between patients brings social connection, comfort, and a form of mutual understanding that is hard to verbalise.  *“So having this weekly activity is already good physically, but it also allows you to see other people when you’re socially isolated.”* (P4)  *“Because we understand each other without talking. We can recognise the one who’s in pain very well. We know that if she stops, there’s a reason, if she doesn’t talk, there’s a reason and if she wants to talk, there’s a reason.”* (P10)  *“I think the impact they have on each other is really strong.”* (S4)  Finally, peer support is identified as a possible resource in the care pathway: it would allow the sharing of experiences, encouragement of other women, and even active involvement in support.  *“I can stop by and talk with women, just explain to them that it feels good and that they have the same difficulties I had. I can help other women and offer relaxation and breathing classes for free.”* (P6)  *“I think it’s always interesting to have the other side of the story… we have our protocols, our idea of how patients experience things, but that’s not necessarily what they’re actually living.”* (S2)  Altogether, these elements highlight that patient-centred care is not just about individual adjustment, but a broader consideration of her experience, her social ties, and her capacity to become an active partner, alongside professionals, in her own care pathway. |
|  |  | **Need for structured interdisciplinary coordination**  The integration of PA as a routine component of care relies on smooth coordination between hospital and community settings, based on mutual understanding of roles, structured information-sharing and consistency of messages throughout the care pathway.  In hospital settings, the sports medicine department is perceived as a strategic resource. It enables the performance of functional assessments, ensures safe follow-up and reinforces consistency in practice.  *“The sports medicine department is there to show us that it’s fun, that it’s good, that it’s not an obstacle course.”* (P5)  However, silos between hospital departments persist, limiting the visibility of available resources and hindering smooth transitions within the care pathway.  *“I don’t even know if they have an activity dedicated to that.”* (PHY2)  *“I think we’re very siloed inside the hospital. You know what you should do, and then you know what you can do. And I think the knowledge of what we can do isn’t very good.”* (SMP2)  In the community sector, local resources such as general practitioners, private physiotherapists, or associations offering APA play an essential role.  *“When the GP has already mentioned it to them, it helps us a lot.”* (CCN2)  *“I didn’t have any APA classes near me since I live a bit far from Lyon. So, I found a physiotherapist and started Pilates.”* (P2)  Nevertheless, several barriers remain, such as a lack of awareness of available resources and a lack of structured connection between hospital-based and community professionals.  *“Maybe at least a little summary to give to the physio, so they know what they can do.”* (P3)  *“There are a lot of ‘sport-health’ centres being developed; eventually there might be more suitable, maybe more accessible, options, I don’t know.”* (SMP2)  In response to these identified obstacles, health professionals suggest several areas for improvement. Building networks and organising regular opportunities for exchange are mentioned as concrete levers to avoid fragmentation and strengthen PA integration into care.  *“First, we need to create partnerships, a care network.”* (S1)  *“We need a connection with nearby sports associations, because that helps maintain activity after rehabilitation.”* (SMP2) |
|  |  | **Key role for the APN: APN identified as a potential actor in PA support**  **Health system related factors: helpers and hindrances**  APNs, recently introduced into the hospital landscape, are mentioned by several healthcare professionals as key resources for supporting patients toward PA. Their role is discussed at various points along the care pathway.  During the initial diagnosis consultation, surgeons report that they rarely address PA, mainly due to the large amount of information that needs to be explained and the emotional context experienced by patients.  *“In fact, I don’t really talk about it at the diagnosis consultation, because I already give them quite a lot of information at that point.”* (S1)  In this context, setting up a post-diagnosis consultation led by the APN is seen as an opportunity to revisit the information given during the diagnosis consultation and to address supportive care, including PA. This suggestion is made by professionals both with and without access to an APN in their structure. It is presented as a complementary consultation, distinct from the one conducted by the care coordinator nurse.  *“It’s more during the APN consultation that we really manage to cover the whole world of physical activity.”* (S3)  *“If the APN did a post-diagnosis consultation. I’m not saying I want to remove the nurse-led consultation, but rather that there could be another appointment, more medical, a synthesis where the patient could go over everything and talk about supportive care, including PA.”* (HN)  The APN is also cited as a professional involved in referring patients to professionals who deliver PA programs.  *“The referral, it’s mainly the APN.”* (CCN1)  They are also mentioned among the professionals who can raise patients’ awareness of PA and its benefits, alongside physiotherapists and sports medicine physicians.  *“It could be a physio, a sports doctor, or the APN who could raise their awareness of PA.”* (PHY1)  Moreover, their role, perceived as more accessible than that of doctors, helps facilitate dialogue for some patients.  *“There’s a connection that gets made. The doctor is high in the hierarchy, while women will listen more to nurses, they’ll let themselves be guided more. They’ll be less on the defensive.”* (P10)  Finally, in the structures where an APN is present, this professional is seen as a resource to help train other caregivers, particularly junior doctors.  *“It would be good to create a specific training. The APN could create a more specific training, for example, for interns in the department.”* (S2)  While organizational and human adjustments appear essential, other potential levers such as digital tools are also mentioned to help facilitate the integration of PA into the care pathway. |
|  | **Underuse of digital tools** | **Structuring the Initial assessment of PA : observations and potential contributions of digital tools**  Patients and healthcare professionals expressed a set of expectations and perspectives regarding the use of digital tools in the integration of PA within the care pathway. Three complementary dimensions emerged from the verbatim accounts: the absence of digital structuring of the initial PA data collection, the still occasional use of digital information supports and the prospects for using e-health applications.  A heterogeneity in practices was observed regarding the collection of PA information during consultations. Several professionals interviewed pointed out the absence of a structured collection process.  *“I always ask them what kind of physical activity they do and I write it down.”* (S2)  *“I get the feeling that if patients don’t bring up the subject, we won’t necessarily go into it in much detail, we’ll just tell them ‘it’s good to do a little sport.’”* (S4)  Patients confirm this variability, with some reporting a complete lack of information and others describing multiple professionals bringing up the subject without any apparent coordination.  *“I’ve never been sporty and nobody talked to me about physical activity during my care.”* (P15)  *“They all asked me questions: oncologist, APN, surgeon.”* (P5)  This lack of structure can lead to a delayed commitment to engaging in PA.  *“I would have started earlier if I had known.”* (P14)  In response, several professionals suggest integrating PA into the standard clinical assessment, in the same way as medical history.  **Health system related factors: helpers and hindrances**  *“It should be something we talk about kind of systematically, like medical history, ask if she does sport. Like with tobacco.”* (S3)  In this perspective, digital tools (such as standardized items in patient records or questionnaires to be completed) are mentioned as potential ways to facilitate this data collection.  *“We should change our digital report template and add items about PA after the medical history or have them fill out questionnaires.”* (S4)  The aim of these adjustments is to harmonize practices and facilitate long-term follow-up.  *“We should think about harmonizing our practices on this topic.”* (MO2)  However, time constraints during consultations are often cited as a barrier to adding new tools or routines.  *“We need to keep in mind that we only have 20 minutes per consultation.”* (S2)  Digital technology appears as a potential area for improvement, but one that remains to be explored, considering organizational realities and the feasibility in routine clinical practice. |
|  |  | **Lack of practical knowledge to integrate PA into daily life: between human support and digital communication tools**  The interviews reveal a marked heterogeneity in the patients’ levels of knowledge about the benefits, risks, or concrete ways of practicing PA during their care pathway.  *“And for me, I would have liked it to be explained well before I had to start repairing the damage.”* (P14)  Patients and healthcare professionals agree on the need for a progressive educational framework, combining information, experimentation and concrete appropriation.  *“At the hospital, I would have liked to really see the body as a student, like here is this muscle, here is that muscle, the shoulder blades, the collarbones, the shoulder for example. You need to bring knowledge.”* (P6)  *“Sometimes, they’re not even aware they’re sedentary or inactive. So, they’re not aware that it’s a problem.”* (SMP1)  Healthcare professionals emphasize that this information must be personalized, considering the patient’s health literacy level, previous experience with PA, personal values, life priorities and more broadly, her overall history. The goal is to co-construct accessible knowledge that can be transferred into everyday life and supports the patient’s ability to act.  *“If we don’t consider our personal baggage, case by case, it doesn’t work, it won’t work, it blocks at some point.”* (P5)  *“We take the patient where she is, with an initial assessment, and we will bring her along and propose relevant and adapted goals, adaptable to her lifestyle.”* (APN1)  Hybrid formats are proposed: friendly group sessions, digital materials (podcasts, videos), or information displayed in care settings.  *“I would see it like setting a meeting with several women, like a class reunion. Around a little coffee, talk to them about sport and the good it can do them.”* (P13)  *“After the surgeon announced the cancer, it would be nice to have a nurse who gives you a list of recommendations with podcasts to listen to, videos to watch.”* (P9)  These two approaches are also favoured by healthcare professionals.  *“To have a relaxed space for discussions and meetings.”* (S3)  *“Why not simply share the information in a video format in waiting rooms.”* (APNS)  Among the digital proposals, podcasts are particularly appreciated for their ease of access, intimate feel and their potential to offer content that is both educational, inspiring, and reassuring.  *“Listening to a podcast is often something we do for ourselves. It’s a personal act, it’s not imposed.”* (S2)  In parallel, they express a need for ongoing training, more practical than theoretical, to better guide, prescribe, and motivate.  *“It’s the information for teams and patients that will improve overall care and bring PA into people’s routine.”* (MO1)  *“We don’t have hands-on courses on how to prescribe, how to refer. On the other hand, we are well informed about the importance of PA during the care pathway.”* (S4)  *“We inform, but do we explain and give it meaning? That, I think we’re not good at.”* (HN)  Hybrid training formats, combining in-person sessions and recorded materials, are mentioned to ensure flexible access to information.  *“It would be good to have in-person trainings, but also recorded ones, so we can watch them again or access them if we can’t attend.”* (PHY1) |
|  |  | **Lack of virtual coaching for sustainable and tailored follow-up**  The use of digital tools appears to be a promising lever to support engagement in PA, both for patients and healthcare professionals. These tools can act as sources of information, help establish routines and serve as motivation.  Participants mention a growing interest in flexible formats that make information more accessible, support engagement in tailored practices and help maintain regularity despite daily life constraints.  *“A little thing that sends you small reminders, little pop-ups, because sometimes they just don’t think about it, really, they have other priorities in life.”* (S3)  *“An app on your phone, with simple exercises depending on your abilities, that you can do if you're at work.”* (MO2)  However, several participants emphasized that these tools should not be designed as normative or guilt-inducing systems, but rather as flexible resources that adapt to each patient’s rhythm, capacities and preferences.  *“I think having a tool that lets you set routines daily is better than something that tells you: ‘you didn’t reach your step count today’. It’s much less guilt-inducing.”* (S3)  *“I don’t want to put pressure on them, I don’t want them to feel attacked or monitored.”* (PHY1)  Far from being prescriptive, the effectiveness of these tools relies on their personalization, the variety of content (nutrition, relaxation, yoga, etc.), and their grounding in the real-life experiences of the women concerned.  *“On a blog, we could bring in healthcare professionals, patients, but also yoga or dance instructors. The idea would be to combine medical, educational and experiential approaches.”* (S3) |
| APAI: Adapted Physical Activity Instructor; APN: Advanced Practice Nurse; APNS: Advanced Practice Nurse Student; CCN: Care Coordinator Nurse; HN: Head Nurse; MO: Medical Oncologist; P: Patient; PA: Physical Activity; PHY: Physiotherapist; SPM: Sports Medicine Physician; S: Surgeon | | |
